# Predictive Values of Serum *Chlamydia trachomatis* TroA and HtrA IgG Antibodies as Markers of Persistent Infection in the Detection of Pelvic Adhesions and Tubal Occlusion

**DOI:** 10.3390/microorganisms7100391

**Published:** 2019-09-25

**Authors:** Tiina Rantsi, Jolande A. Land, Päivi Joki-Korpela, Sander Ouburg, Kati Hokynar, Jorma Paavonen, Aila Tiitinen, Mirja Puolakkainen

**Affiliations:** 1 Department of Obstetrics and Gynecology, University of Helsinki and Helsinki University Hospital, PO Box 140, FI-00029 HUS, Helsinki, Finland; paivi.joki-korpela@hus.fi (P.J.-K.); jorma.paavonen@helsinki.fi (J.P.); aila.tiitinen@helsinki.fi (A.T.); 2 Institute for Public Health Genomics (IPHG), Department of Genetics and Cell Biology, Research Institute GROW, Faculty of Health, Medicine & Life Sciences, University of Maastricht, 6229 ER Maastricht, The Netherlands; j.land@maastrichtuniversity.nl (J.A.L.); s.ouburg@amsterdamumc.nl (S.O.); 3 VU University Medical Center, Department of Medical Microbiology & Infection Control, Laboratory of Immunogenetics, 1081 HZ Amsterdam, The Netherlands; 4 Department of Virology, University of Helsinki and Helsinki University Hospital, FI-00014 Helsinki, Finland; kati.hokynar@helsinki.fi (K.H.); mirja.puolakkainen@helsinki.fi (M.P.)

**Keywords:** *Chlamydia trachomatis*, tubal factor infertility, laparoscopy, *Chlamydia trachomatis* IgG antibody testing

## Abstract

*Chlamydia trachomatis* IgG antibody testing (CAT) has been used as a screening test for tubal factor infertility (TFI), but as the CAT is only a marker of a past exposure to *C. trachomatis* and not of late sequelae, the positive predictive value (PPV) of the test is low. The persistence of *C. trachomatis* in the upper genital tract has been suggested as one of the key mechanisms in the development of TFI. Serum antibodies against *C. trachomatis* TroA and HtrA, proteins expressed specifically during persistent infection, have been suggested as novel biomarkers for TFI diagnostics. We studied serum IgG antibody responses against *C. trachomatis* TroA, HtrA and MOMP in 79 subfertile women, of whom 28 had laparoscopically proven TFI. We confirmed that the accuracy of CAT in diagnosing TFI is low, whereas TroA IgG and HtrA IgG are more accurate tests in detecting tubal occlusion and pelvic adhesions. However, the sensitivity and negative predictive value (NPV) of TroA IgG and HtrA IgG are still too low to justify their use as a screening test in clinical practice. Individual immunogenetic profiles combined with TroA and HtrA antibody responses might identify women with the highest risk for developing late complications after *C. trachomatis* infection.

## 1. Introduction

*Chlamydia trachomatis* infection is the most prevalent bacterial sexually transmitted infection with over 130 million new cases reported annually [1]. The majority of infected women have uncomplicated lower genital tract infections, but some women develop a persistent or ascending infection [2]. In the latter, infection can lead to severe reproductive morbidity, including tubal factor infertility (TFI) [3]. Tissue damage resulting from chlamydial infection has been attributed to inflammatory processes in the upper genital tract, leading to pelvic adhesions and scarring of the tubal epithelium [4].

Laparoscopy and dye chromopertubation have remained the gold standard for assessing tubal patency and pelvic adhesions in subfertile women [5]. Laparoscopy, however, is an invasive and expensive procedure requiring general anesthesia. Hysterosalpingography (HSG) and hysterosalpingosonography (HSSG) are alternative less-invasive methods for tubal evaluation, but less accurate in identifying TFI, as peritubal adhesions cannot be detected [6].

Women with TFI have serum antibodies against *C. trachomatis* more often than women without tubal pathology [7,8]. Therefore, *C. trachomatis* IgG antibody testing (CAT) has been introduced as a screening test for TFI in the infertility work-up, to select high-risk patients for laparoscopic tubal evaluation [9]. The most commonly used CAT is based on an antibody response to *C. trachomatis* major outer membrane protein (MOMP). The major limitation of CAT in TFI prediction is its low specificity, leading to a low positive predictive value (PPV) [10]. Positive CAT is only a marker of past exposure, but does not reflect persistence or clearance of infection [7].

The persistence of *C. trachomatis* in the upper genital tract has been suggested as one of the key mechanisms in the development of Fallopian tube scarring [11]. *C. trachomatis* TroA and HtrA are essential proteins expressed particularly during persistent chlamydial infection [12,13]. The levels of TroA (YtgA) have been shown to increase when *C. trachomatis* is cultured under iron-starvation conditions, which is one means to induce persistence [14]. High temperature requirement protein (HtrA) plays a role during *C. trachomatis* replication. HtrA levels has been shown to increase during the developmental cycle when *C. trachomatis* is cultured at the presence of penicillin, which is another means to induce persistence in vitro [14]. Also in vivo, IgG antibody responses to *C. trachomatis* TroA and HtrA are more common in patients with ascending and repeated chlamydial infections than in healthy controls [15], and women with TFI have more often serum IgG antibodies to TroA and HtrA than subfertile women with patent Fallopian tubes [16]. Use of such differentially expressed *C. trachomatis* antigens as TroA and HtrA may offer a means to develop more specific tools for TFI diagnostics.

Our goal was to evaluate the performance of *C. trachomatis* TroA and HtrA antibody testing in detecting *C. trachomatis*-related Fallopian tube pathology and pelvic adhesions using laparoscopy as the reference standard, and to compare their accuracy with the most commonly used CAT (MOMP IgG).

## 2. Materials and Methods

### 2.1. Study Population

**Figure 1:**
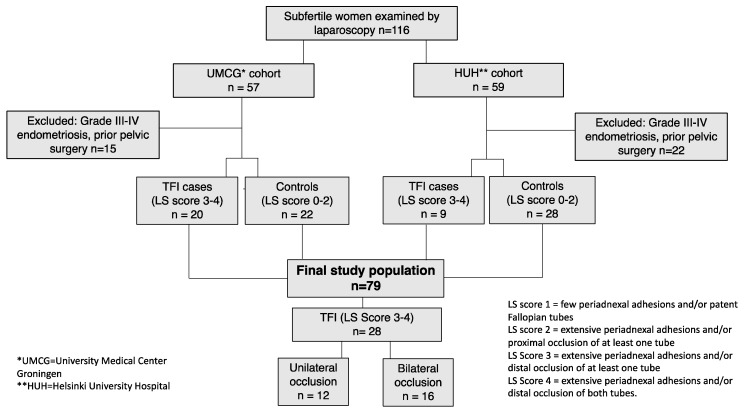
Flowchart of the number of patients (n) in the study.

This study was performed among 116 subfertile women who had undergone laparoscopy and tubal testing with methylene blue dye as part of their infertility evaluation: 57 Dutch women who were part of a cohort of consecutive patients visiting the Fertility Clinic of the University Medical Center Groningen (UMCG) between 2007–2016, and 59 Finnish women who visited the Reproductive Medicine Unit, Helsinki University Hospital (HUH), between 2007–2010 (Figure 1). All participants were Caucasian and had a history of at least one year of subfertility. In the Dutch cohort, laparoscopy was performed in women who had positive CAT results, or when tubo-ovarian abnormalities were found by ultrasound. In the Finnish cohort, laparoscopy was performed in case of severe dysmenorrhea, suspicion of deep rectovaginal endometriosis on physical examination, or a large endometrioma or an ovarian cyst requiring surgical treatment. Bilateral tubal occlusion found by HSSG was confirmed by laparoscopy in the Finnish cohort.

Serum samples of the Dutch women were collected at the first clinic visit as a part of their routine infertility work-up. Spare serum was cryopreserved at −20 °C for research purposes and thawed to perform serological assays for the present study. Serum samples of the Finnish women had been collected and analyzed for our previous studies, in which we studied the performance of *C. trachomatis*-specific cell-mediated and humoral immune responses in TFI prediction [17] and the prevalence of TroA and HtrA antibodies among unselected subfertile women [16].

The laparoscopic findings were retrospectively collected from the patient registries of UMCG and HUH, and scored in a standardized way by two gynecologists (T.R. and J.L.). TFI was classified according to increasing severity of tuboperitoneal adhesions and the patency of the Fallopian tubes (LS-score 0, 1, 2, 3, 4) [18]:

0 = no abnormalities

1 = few periadnexal adhesions and/or patent Fallopian tubes

2 = extensive periadnexal adhesions and/or proximal occlusion of at least one tube

3 = extensive periadnexal adhesions and/or distal occlusion of at least one tube

4 = extensive periadnexal adhesions and/or distal occlusion of both tubes

The laparoscopic findings of endometriosis were graded according to the American Fertility Society classification [19]. Exclusion criteria for the present study were stage II-III (moderate) or IV (severe) endometriosis (*n* = 32), or a history of pelvic surgery other than uncomplicated appendectomy or Caesarean section (*n* = 5). For the present study, TFI was defined as extensive periadnexal adhesions and/or distal occlusion of at least one tube (LS score 3–4), since this has been shown to reflect chlamydia associated tubal pathology most accurately [18]. Subfertile women without TFI (LS score 0–2) served as controls. The controls had unexplained infertility, mild male factor infertility, mild endometriosis, ovulatory disorders or proximal tubal occlusions.

The final study population consisted of 79 subfertile women (42 Dutch and 37 Finnish women) (Figure 1).

### 2.2. Serological Methods

IgG antibody responses to recombinant *C. trachomatis* TroA and HtrA were analyzed by an in-house enzyme immune assay (EIA) at the Department of Virology, Helsinki University, as described earlier [15,16]. Purified TroA (CT067) and HtrA (CT823) proteins were coated (100 ng) to Nunc Maxisorp ELISA plate wells. After coating, the wells were washed and blocked with 3% BSA. Sera diluted 1:200 were tested, and binding was detected with 1:1000 diluted polyclonal anti-Human IgG-HRP (Dako, Glostrup, Denmark) and 3,3’,5,5’-tetramethylbenzidine substrate (TMB, Labsystems Diagnostics, Vantaa, Finland). Each serum sample was analyzed in duplicate in antigen-coated and non-coated wells, and the absorbance value of the non-coated well was subtracted from the absorbance value of the antigen-coated well. A positive serum (serum reactive with the purified protein in western blotting) and a negative serum as well as buffer-only wells were included in each run. The cut-off values for the assay were based on the absorbance values (mean + 2 SD) obtained from specimens of sexually inexperienced girls, not exposed to *C. trachomatis* (mean age 12 years, range 8–17). TroA antibody was detected in 8.1% (mean absorbance at 450 nm 0.216) of sera from healthy blood donors, HtrA antibody in 5.4% (mean absorbance at 450 nm 0.113).

*C. trachomatis* MOMP-specific IgG antibody response had been analyzed as a part of the routine infertility work-up in the Dutch cohort, and as part of our previous study in the Finnish cohort [17]. The serological assay for MOMP IgG detection used in both cohorts was a commercially available ELISA kit (Medac Diagnostica, Hamburg, Germany). Sera were diluted 1:100 and tested according to the manufacturer’s instructions. The results were expressed as a mean absorbance at 450 nm. The cut-off value for a positive antibody result (mean optical density [OD] of the negative control +0.350) was OD > 0.4.

### 2.3. Statistical Methods

Characteristics of women with and without TFI were compared using the Mann-Whitney *U*-test and *X*^2^-test. For comparison of the prevalence of *C. trachomatis* antibodies in women with and without TFI, *X*^2^-test was used. *X*^2^-test for trend and Kruskal-Wallis test were used to compare *C. trachomatis* TroA, HtrA, and MOMP seroprevalences and to study TroA and HtrA IgG absorbance levels in relation to the severity of TFI. The prognostic value of *C. trachomatis* antibody tests for TFI was analyzed by calculating the sensitivity, specificity, accuracy, positive predictive value (PPV), and negative predictive value (NPV) for single tests and test combinations. Statistical analyses were performed by IBM SPSS Statistics 25.0 (IBM Corp., Armonk NY, USA). *p*-value < 0.05 was considered statistically significant.

### 2.4. Ethical Approval

Dutch women attending the UMCG Fertility Clinic had approved the use of their anonymized medical data and spare serum samples for research purposes. The procedure was approved by the medical ethical board of the Amsterdam University Medical Centers (#10.17.0046, 16/02/2016). Use of serological and medical data of Finnish women was approved by the Helsinki University Hospital Ethical Committee (Dnro 29/E9/07), 19 March 2007).

## 3. Results

### 3.1. Tubal Factor Infertility (TFI) in the Study Population

In our selected study population, altogether 28 (35.4%) of the 79 women fulfilled the definition of TFI (i.e. extensive peritubal adhesions and/or distal occlusion of at least one Fallopian tube). Tubal occlusion was bilateral in 16 (57.1%) cases and unilateral in 12 (42.9%) cases (Figure 1). The mean age (31.1 vs. 31.1 years, respectively) and the duration of subfertility (2.5 vs. 2.6 years, respectively) were comparable between women with and without TFI.

### 3.2. Serological Results

Of the 79 women, 28 (35.4%) had *C. trachomatis* TroA IgG antibodies, 27 (34.2%) had HtrA IgG antibodies, and 32 (40.5%) had IgG antibodies against *C. trachomatis* MOMP. Women with TFI had more often TroA IgG (60.7% vs. 21.6%, *p* < 0.001) and HtrA IgG antibodies (57.1% vs. 21.6%, *p* = 0.001) than women without TFI (Table 1). The difference in the presence of MOMP IgG antibodies between TFI and non-TFI groups was not statistically significant (53.6% vs. 33.3%, *p* = 0.08).

Table 2 shows the performances of single antibody tests and test combinations for TFI. *C. trachomatis* TroA IgG antibody test was the best single test in detecting TFI, with an accuracy of 72.2%, sensitivity of 60.7% and specificity of 78.4% (Table 2). PPV of TroA IgG (in our study population with 35.4% TFI prevalence) was 60.7% and NPV 78.4%. Specificity increased to 86.3% when the combination of TroA and HtrA IgG antibodies was used, but sensitivity decreased to 53.6%. MOMP IgG showed a specificity of 66.9% and sensitivity of 53.6% for the detection of TFI. Combining MOMP antibodies with TroA IgG or HtrA IgG increased specificity to 88.2%, but sensitivity of the combination was 35.7%.

### 3.3. Serum Antibody Levels by the Severity of TFI

**Figure 2:**
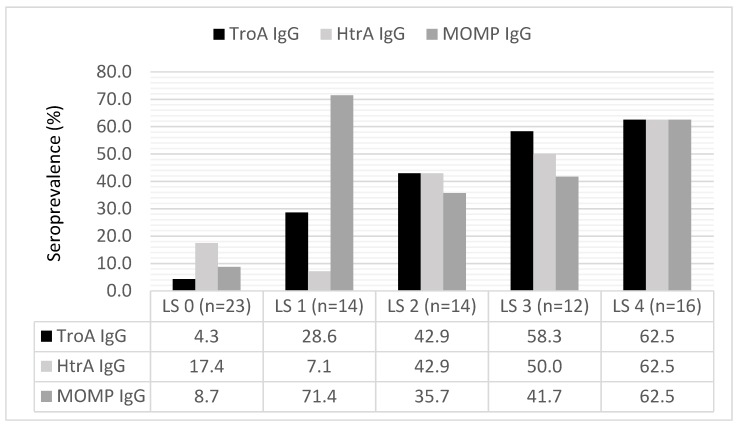
The prevalence (%) of *C. trachomatis* TroA IgG, HtrA IgG, and MOMP IgG antibody by the severity of TFI. The severity of pelvic adhesions was graded by the laparoscopy score (LS-score) scale modified from Land et al., 1998 [18]: 0 = no abnormalities, 1 = few periadnexal adhesions and/or patent Fallopian tubes, 2 = extensive periadnexal adhesions and/or proximal occlusion of at least one tube, 3 = extensive periadnexal adhesions and/or distal occlusion of at least one tube, 4 = extensive periadnexal adhesions and/or distal occlusion of both tubes.

The prevalence of *C. trachomatis* TroA IgG (*p* < 0.001) and HtrA IgG (*p* = 0.001) antibodies increased by increasing severity of TFI (Figure 2). Also, the mean absorbance level (A_450nm_) of TroA IgG, and HtrA IgG antibodies increased by increasing severity of TFI, being highest in the LS-score group 4 (mean A_450nm_ [SD, range]) 1.54 [1.54, 0.07—3.83]) for TroA and 1.41 [0.007—3.81]) for HtrA).

## 4. Discussion

The aim of this study was to evaluate the performance of *C. trachomatis* TroA IgG and HtrA IgG in diagnosing TFI, and to compare them to the most commonly used CAT (MOMP IgG). We found that TroA and HtrA antibodies were strongly associated with TFI, but the antibody response against *C. trachomatis* MOMP did not show this association. This is in agreement with several earlier studies that have observed a relatively low diagnostic accuracy of MOMP IgG in TFI prediction [8,9,17,20]. MOMP is a protein accounting for the majority of the outer membrane of the bacterium [14]. Antibody response against MOMP reflects previous exposure to the pathogen, but does not provide information about clearance or persistence of *C. trachomatis* [7]. Our study confirms the hypothesis that particularly prolonged exposure to *C. trachomatis* leads to chronic inflammatory responses and reproductive sequelae, as TroA and HtrA are specific *C. trachomatis* proteins produced during persistent stages of chlamydial infection.

We were the first to evaluate the performance of *C. trachomatis* TroA and HtrA antibody tests in subfertile women, by using laparoscopic results as the gold standard for TFI. Ideally, a test with high NPV and high sensitivity could rule out *C. trachomatis*-related TFI and avoid unnecessary and invasive examinations in patients. In our study, we could not identify this ideal test, as the best single test for TFI detection was TroA IgG antibody with only moderate sensitivity (60.7%) and NPV (78.4%). Combining TroA IgG with HtrA or MOMP IgG did not improve the sensitivity of the test combination. The combination of HtrA and MOMP IgG had the highest specificity, but the sensitivity was low because of many false negative test results.

The seropositivity rates and the serum absorbance levels of TroA and HtrA antibodies increased with increasing severity of TFI. This finding is in line with previous studies showing a linear relationship between serum MOMP IgG titers and severity of tubal damage [21,22]. In these studies, high antibody titers were also observed in some women with normal pelvic anatomy, whereas in our study, no patients with normal pelvic anatomy had high TroA IgG and HtrA IgG absorbance values (mean A_450nm_ ≥ 1.0).

The predictive value of CAT for TFI is highly dependent on the definition of TFI and the selection of cases and controls. Our cohort comprised of 79 subfertile women with laparoscopically evaluated tubal status from the fertility centers of two large university hospitals in Europe, and TFI was uniformly defined. However, the retrospective nature of our study has potential sources of bias, including incomplete documentation and the variability in the quality of data reported by laparoscopists. The majority of Dutch women included in the study were selected for laparoscopy based on their initial CAT result, and therefore, more CAT positive women had laparoscopies than CAT negative women. In the Finnish cohort, women were selected for laparoscopy based on their risk for severe endometriosis or abnormalities on HSSG. This selection bias explains the high TFI rate in our study, influencing the predictive values, which are known to be dependent on the prevalence of the disease. We acknowledge the limited number of patients in the final cohort of our study. However, this is an exploratory study focusing on chlamydia-related TFI, and the study population consisted of women with the highest risk of previous chlamydial infection. Although the absolute predictive values may be overestimated, the relative differences between the performances of the tests will be accurate and thus applicable into the study populations with lower TFI prevalence.

Despite the overwhelming evidence that *C. trachomatis* infection is causally linked to salpingitis and TFI, we found the predictive value of TroA and HtrA *C. trachomatis* serology in diagnosing tubal damage and pelvic adhesions to be only moderate. Not every patient with TFI has been infected with *C. trachomatis* or will develop a humoral immune response to *C. trachomatis*. Tubal damage can be caused also by other pelvic infections, such as *Neisseria gonorrhoeae* or *Mycoplasma genitalium* infection [23]. Furthermore, as tubal tissue damage is immune-mediated, the development of TFI is affected by the host immune response [24,25] and the virulence of the causative organism [11,26]. It is also worth noticing that in our study not all subfertile women with positive serum TroA and HtrA IgG antibody tests had tubal damage or pelvic adhesions. This may be explained by the fact that tubal pathology not necessarily appears as visible occlusions. Silent, persistent chlamydial infections may cause non-visible mucosal damage, impairing the function of the tube and the likelihood of natural conception [27]. Thus, it would be interesting to study the time to pregnancy in TroA and HtrA IgG positive patients without visible tubal pathology at laparoscopy.

We confirmed that the accuracy of CAT by MOMP IgG in the prediction of TFI is low, whereas *C. trachomatis* TroA IgG and HtrA IgG are more accurate tests for TFI due to their higher specificity and higher PPV. However, their sensitivity and NPV are still too low to justify their use as a screening test in clinical practice. Chlamydial pelvic infections and TFI are both complex, multifactorial diseases and it seems highly unlikely that one single test would be specific enough to predict TFI as a late consequence of chlamydial infection. Because antibody responses to TroA and HtrA may indicate the course of chlamydial infection more accurately than MOMP IgG, and tissue damage is known to be affected by immunogenetic variation in the host [25], it might be interesting to study TroA and HtrA serology in combination with genetic profiling, to identify women with the highest risk for late complications.

## Figures and Tables

**Table 1:** The prevalence of *C. trachomatis* TroA, HtrA and MOMP IgG antibodies in tubal factor infertility (TFI) cases and controls.

	TFI (*N* = 28)	Controls (*N* = 51)	*p*-Value
TroA IgG (%)	60.7 (17/28)	21.6 (11/51)	0.001
HtrA IgG (%)	57.1 (16/28)	21.6 (11/51)	0.001
MOMP IgG (%)	53.6 (15/28)	33.3 (17/51)	0.08

**Table 2:** Predictive values of single and combinations of antibody tests for detecting TFI with 95% confidence intervals (CI).

Antibody Test	Sensitivity (%, 95% CI)	Specificity (%, 95% CI)	Accuracy (%, 95% CI)	PPV (%, 95% CI)	NPV (%, 95% CI)
TroA IgG	60.7 (40.6⎯78.5)	78.4 (64.7⎯88.7)	72.2 (60.9⎯81.7)	60.7 (45.8⎯73.8)	78.4 (69.2⎯85.5)
HtrA IgG	57.1 (37.2⎯75.5)	78.4 (64.7⎯88.7)	70.9 (59.6⎯80.6)	59.3 (44.1⎯72.9)	76.9 (68.0⎯84.0)
MOMP IgG	53.6 (33.9⎯72.5)	66.7 (52.1⎯79.2)	62.0 (50.4⎯72.7)	46.9 (34.4⎯59.7)	72.3 (62.7⎯80.3)
**Test combinations**					
TroA + HtrA IgG	53.6 (33.9⎯72.5)	86.3 (73.7⎯94.3)	74.7 (63.6⎯83.8)	68.2 (49.8⎯82.2)	77.2 (69.1⎯83.6)
TroA + MOMP IgG	39.3 (21.5⎯59.4)	84.3 (71.4⎯92.3)	68.4 (56.9⎯78.4)	57.9 (38.5⎯75.1)	71.7 (64.7⎯77.7)
HtrA + MOMP IgG	32.1 (15.9⎯52.4)	90.2 (78.6⎯96.7)	69.6 (58.3⎯79.5)	64.3 (40.1⎯82.9)	70.8 (64.9⎯76.0)
TroA + HtrA + MOMP IgG	35.7 (18.6⎯55.9)	88.2 (76.1⎯95.6)	69.6 (58.3⎯79.5)	62.5 (40.4⎯80.4)	71.4 (65.1⎯77.0)

PPV, positive predictive value; NPV, negative predictive value.
